# Expression patterns of two *pal* genes of *Pleurotus ostreatus* across developmental stages and under heat stress

**DOI:** 10.1186/s12866-019-1594-4

**Published:** 2019-10-26

**Authors:** Ludan Hou, Lining Wang, Xiangli Wu, Wei Gao, Jinxia Zhang, Chenyang Huang

**Affiliations:** 1 Institute of Agricultural Resources and Regional Planning, Chinese Academy of Agricultural Sciences, Beijing, China; 2 Key Laboratory of Microbial Resources, Ministry of Agriculture and Rural Affairs, 12 Zhongguancun South Street, Beijing, 100081 China

**Keywords:** *Pleurotus ostreatus*, Phenylalanine ammonia-lyase, Development, Heat stress, Overexpression, RNA interference

## Abstract

**Background:**

Phenylalanine ammonia-lyase (PAL, EC 4.3.1.24) is the first key enzyme in the phenylpropanoid pathway. The *pal* gene has been widely studied in plants and participates in plant growth, development and defense systems. However, in *Pleurotus ostreatus*, the biological functions of *pal* during organismal development and exposure to abiotic stress have not been reported.

**Results:**

In this study, we cloned and characterized the *pal1* (2232 bp) and *pal2* (2244 bp) genes from the basidiomycete *P. ostreatus* CCMSSC 00389. The *pal1* and *pal2* genes are interrupted by 6 and 10 introns, respectively, and encode proteins of 743 and 747 amino acids, respectively. Furthermore, prokaryotic expression experiments showed that PAL enzymes catalyzed the conversion of L-phenylalanine to trans-cinnamic acid. The function of *pal1* and *pal2* was determined by constructing overexpression (OE) and RNA interference (RNAi) strains. The results showed that the two *pal* genes had similar expression patterns during different developmental stages. The expression of *pal* genes was higher in the reproductive growth stage than in the vegetative growth stage. And the interference of *pal1* and *pal2* delayed the formation of primordia. The results of heat stress assays showed that the RNAi-*pal1* strains had enhanced mycelial tolerance to high temperature, while the RNAi-*pal2* strains had enhanced mycelial resistance to H_2_O_2_.

**Conclusions:**

These results indicate that two *pal* genes may play a similar role in the development of *P. ostreatus* fruiting bodies, but may alleviate stress through different regulatory pathways under heat stress.

## Background

Phenylalanine ammonia-lyase (PAL, EC 4.3.1.24) is the first enzyme in the phenylpropanoid pathway and catalyzes the conversion of L-phenylalanine to trans-cinnamic acid by nonoxidative deamination [1–3]. Furthermore, PAL is the first key enzyme in the phenylpropanol pathway, participating in the formation of a series of structural and defensive phenolic compounds, such as lignin, phenolic acid and hydroxybenzoic acid, flavonoids and stilbene in plants [4]. The *pal* gene has been widely studied in plants and participates in plant growth, development and defense systems [5, 6], including lignin synthesis in cell walls, nutrient transport, and the regulation of seed color [7]. Plants can induce PAL enzymes under abiotic stresses (e.g., ultraviolet-B (UV-B) light, high and low temperature, injury, salt.), leading to the accumulation of phenolic compounds such as flavonoids and phenolic acids [8]. Under salt stress, the antioxidant capacity of plants has been shown to be enhanced by increasing PAL activity [9]. Under UV-B stress, the content of salicylic acid in soybean roots and leaves increased with the increase of PAL activity, showing strong stress resistance [10]. The *pal* gene has also been studied recently in mushrooms, such as *Flammulina velutipes*, in which the *pal* gene was cloned and characterized. The different expression patterns of the *F. velutipes pal* gene and its activity in different organs of the mushroom indicated that *pal* is associated with mushroom growth [11]. In *Tricholoma matsutake*, transcriptome analysis revealed a pattern of *pal* gene expression that was dependent on the developmental stage, suggesting that *pal* has many physiological functions in this mushroom [12]. In several basidiomycete fungi, a metabolic pathway for the metabolism of phenylalanine via cinnamic, benzoic, *p*-hydroxybenzoic, and protocatechuic acids has been reported that is similar to that observed in plants [13]. However, the biological function of *pal* in *P. ostreatus* during development and under abiotic stress has not been reported.

*Pleurotus ostreatus* is one of the most widely cultivated mushroom species globally [14], being an edible mushroom with high nutritional and medicinal value. The mechanism of fruiting body development of edible fungi has been a popular research topic in recent years, with numerous studies investigating fruiting body development and associated signaling pathways. A number of functional genes, such as nicotinamide adenine dinucleotide phosphate oxidase [15], cytochrome P450 [16], superoxide dismutase [17], multicopper oxidases [18] and catalase [19], have been identified and characterized with respect to mushroom development. In addition, the AMP signaling pathway has been reported in mushrooms [20]. The development of mushrooms is a complex process that is regulated by gene products and environmental factors. In China, *P. ostreatus* is primarily cultivated in horticultural facilities, thus its cultivation is strongly affected by seasonal temperature changes, especially the summer high temperatures. A number of studies have shown that high temperatures can affect mycelial growth and fruiting body development [21], and can even lead to spawn burning [22] and *Trichoderma* contamination [23]. Previous studies on the heat stress response of *P. ostreatus* have investigated programmed cell death [24], the role of catalase in fruiting body development and heat stress [19], and the effect of trehalose on mycelial damage mitigation [25]. Recently, Zou et al. studied proteome changes in *P. ostreatus* mycelia during heat stress and recovery and identified 204 proteins, including PAL, exhibiting altered expression during heat stress or the recovery phase [26]. These finding laid a foundation for studying the biological functions of *pal* in *P. ostreatus* under heat stress.

Many studies have used molecular and genetic methods to silence the *pal* gene to study its biological functions in plant growth, development and environmental stress [7, 27]. In recent years, RNA interference (RNAi) and Overexpression (OE) technologies have been widely used to study gene functions in *P. ostreatus*. For example, the overexpression of a methionine sulfoxide reductase A gene enhances stress tolerance in *P. ostreatus* [28], which provides a more effective method for studying the function of genes in *P. ostreatus*. In this study, we searched and cloned the *pal* genes from the *P. ostreatus* genome. On the basis of describing their characteristics, we studied the role of *pal* genes in fruiting body development and heat stress using RNAi and OE technologies.

## Results

### Cloning and bioinformatics analysis of *pal*

**Fig. 1:**
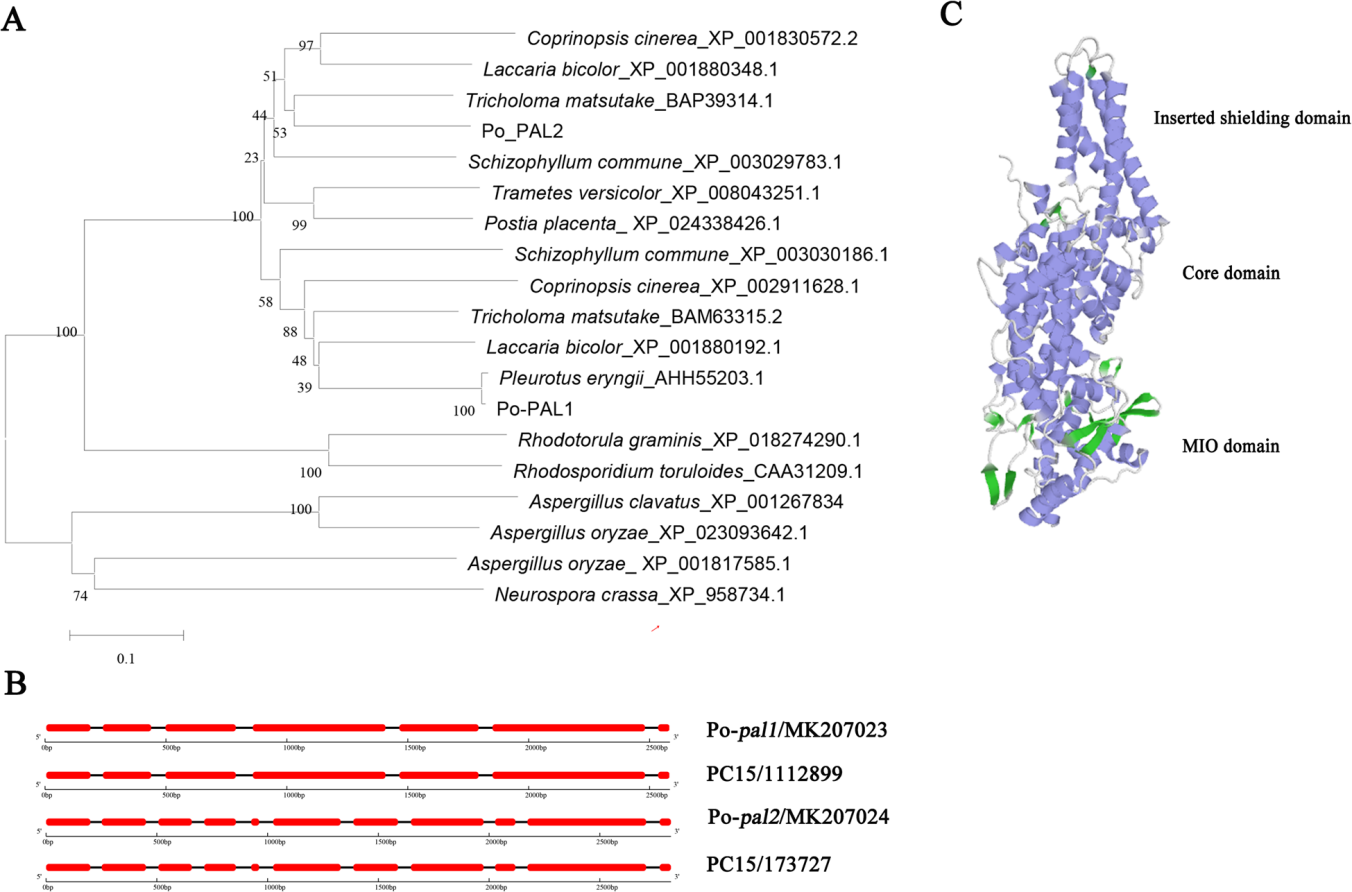
Relationships of fungal PALs, gene structural features and a 3-D structural model. **a** A neighbor-joining phylogenetic tree of PAL protein sequences from 11 fungal species. **b** Gene structures of selected *pal* genes in *P. ostreatus* CCMSSC00389 and PC15. The exons are represented by red rectangles, and the black lines connecting two exons represent introns. **c** 3-D structural model of the Po-PAL protein. The structure was divided into three parts: the 4-methylideneimidazole-5-one (MIO) domain, the core domain and the inserted shielding domain. The MIO group is highlighted in green

Two *pal* genes were identified in the *P. ostreatus* genome and were named *pal1* and *pal2*, respectively. Their full-length cDNA sequences were 2232 and 2244 bp, respectively. DNA sequence analysis showed that 7 exons are interrupted by 6 introns in *pal1*, while 11 exons are interrupted by 10 introns in *pal2* (Fig. 1b). The two sequences were deposited in GenBank with the accession numbers MK207023 and MK207024, respectively. Figure 1b shows that *pal1* has an identical gene structure with the genes encoding PC15_1111887 and that *pal2* has an identical gene structure with the gene encoding PC15_173,727. The PAL1 and PAL2 protein sequences in both *P. ostreatus* CCMSSC 00389 and PC15 were highly similar to one another (Additional file 1: Figure S1), and the consistency reached 99.73 and 99.59%, respectively. However, the amino acid sequences and nucleotide sequences of *pal1* and *pal2* had low similarity in *P. ostreatus* CCMSSC 00389 (Additional file 1: Figure S1 and Additional file 2: Figure S2).

To understand the phylogenetic relationship between the PAL proteins and other fungal PALs, phylogenetic analysis was performed. Phylogenetic analysis of 19 PAL sequences revealed two distinct branches (Fig. 1a). The phylogenetic tree showed that PAL1 and PAL2 have higher similarities to the protein sequences of other mushrooms or fungi than to each other. The cladogram revealed the variation in the PAL protein sequence among fungi.

**Fig. 2:**
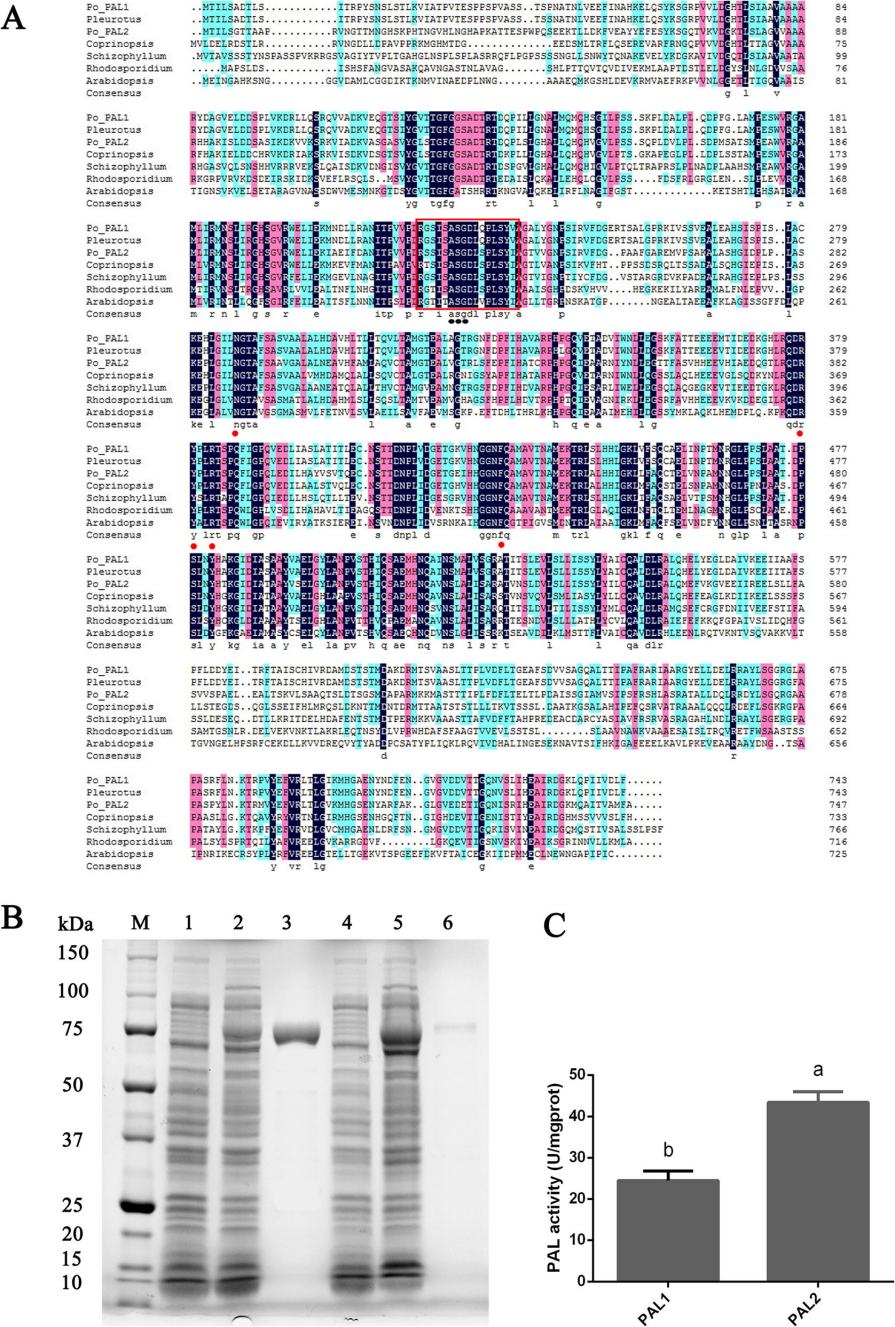
Partial amino acid sequence alignment and SDS-PAGE results of *pal1* and *pal2* expressed in *E. coli*. **a** Partial amino acid sequence alignment of PAL1 and PAL2 of *P. ostreatus* CCMSSC00389 and other PALs. Positions with identical amino acid residues are indicated by circles below the sequence. (*Pleurotus eryngii*_AHH55203.1, *Schizophyllum commune*_XP_003030186.1, *Coprinopsis cinerea*_XP_001830572.2, *Rhodosporidium toruloides*_CAA31209.1, *Arabidopsis thaliana*_NP_181241.1). **b** SDS-PAGE analysis of recombinant PAL protein extracted from *E. coli* BL21 (DE3) cells. M, protein molecular weight standards; 1, crude lysate of *pal1* from *E. coli* BL21 (DE3) grown at 16 °C for 12 h; 2, crude enzyme of *pal1* from *E. coli* BL21 (DE3)/pSMART-V-PAL induced with IPTG (1 mM) at 16 °C for 12 h; 3, *pal1* protein purified with a nickel column; 4, crude lysate of *pal2* from *E. coli* BL21 (DE3) grown at 16 °C for 12 h; 5, crude enzyme of *pal2* from *E. coli* BL21(DE3)/pSMART-V-PAL induced with IPTG (1 mM) at 16 °C for 12 h; and 6, *pal2* protein purified with a nickel column. **c** Determination of PAL activity. Three independent biological replicates were performed for all experiments. The values are the means ± SE. Different letters indicate significant differences between the strains (*P* < 0.05, according to Tukey’s test)

The *pal1* and *pal2* sequences were bioinformatically analyzed to determine their physicochemical properties and possible structure. The *pal1* gene encodes a putative 743-amino-acid polypeptide with an approximate molecular weight and calculated pI of 79.845 kDa and 5.28, respectively. The *pal2* gene encodes a putative 747-amino-acid polypeptide of 79.946 kDa with a predicted isoelectric point of 6.11 [29]. An online analysis revealed a Pfam lyase aromatic domain in both *pal1* and *pal2*, whereas only *pal2* was observed to have a SCOP d1qj5a_domain. SCOP d1qj5a_domain starts at amino acid position 638 and ends at position 735 and it belongs to the PLP-dependent transferase superfamily. The gene models for *pal1* and *pal2* from different organisms are shown in Fig. 2a, which primarily describes the amino acid identities and similarities among *pal* genes in different organisms. The *pal* motif is labeled with a red box, and the conserved active-site motif (Ala-Ser-Gly) and specific amino acids are also shown in Fig. 2a. The conserved active-site motif is labeled with circles under the specific amino acids, which can be converted into an MIO (4-methylidene-imidazole-5-one) prosthetic group (Fig. 1c). The other active-site residues are labeled with red circles in Fig. 2a. The 3-D structure of PAL (Fig. 1c) showed that it is composed of an MIO domain, a core domain and an inserted shielding domain [30]. Thus, the PAL amino sequences are highly conserved with other characterized PAL proteins in fungi.

The analysis of prokaryotic expression showed that the purified proteins (PAL1 and PAL2) had molecular weights of approximately 75 kDa (Fig. 2b), which is consistent with predictions. The activity of the purified enzymes was determined by spectrophotometry (Fig. 2c). The results showed that the activity of PAL1 (24.469 ± 2.296 u/mg protein) was significantly lower than that of PAL2 (43.387 ± 2.551 u/mg protein).

### Expression of *pal1* and *pal*2 during different *P. ostreatus* developmental stages

**Fig. 3:**
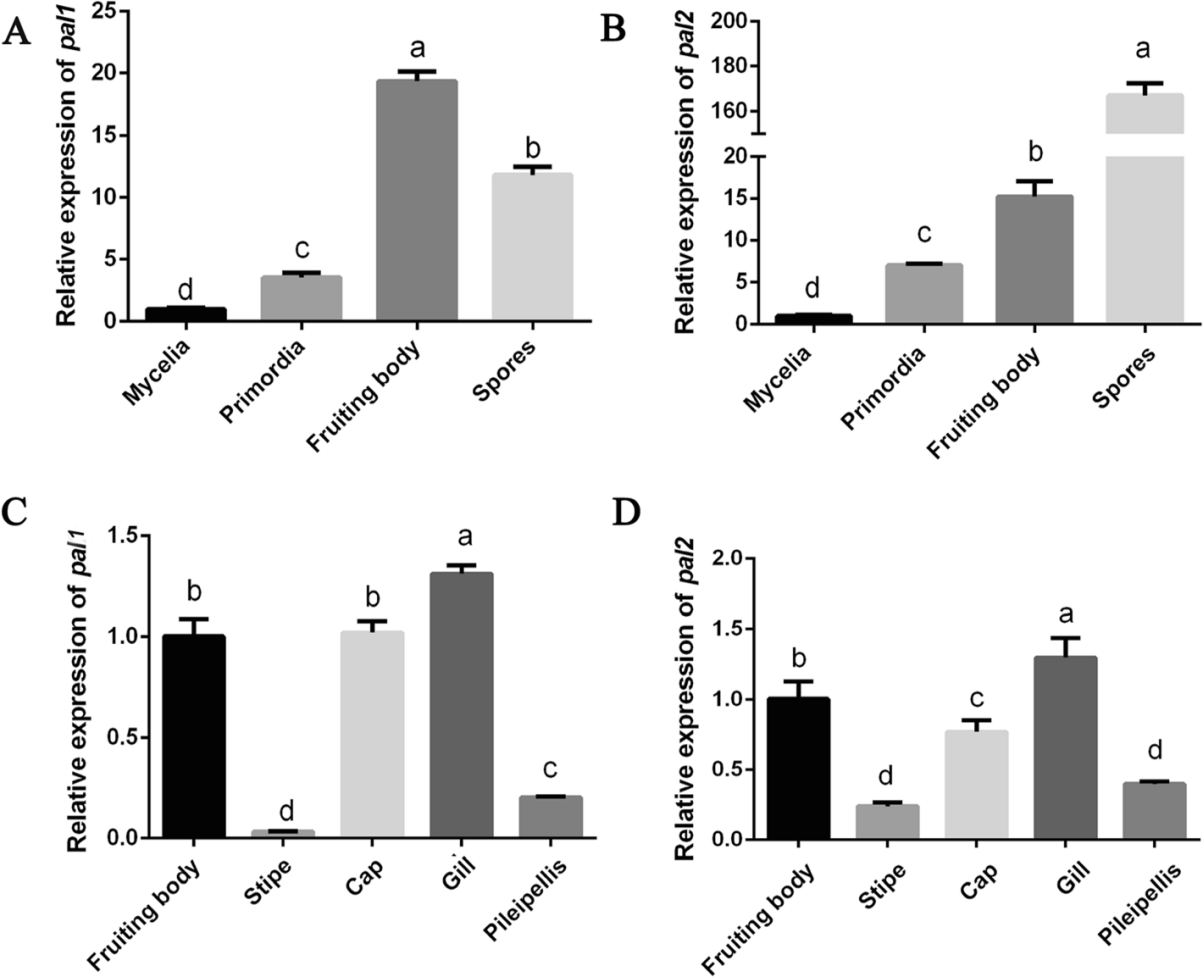
The expression of *pal* during different developmental stages and different parts of *P. ostreatus* fruiting bodies. **a** qPCR analysis of *pal1* expression in WT strain during different cultivation stages. **b** qPCR analysis of *pal2.* The relative abundances of the WT transcript levels at different stages were normalized by comparison with that observed in the mycelium stage (relative transcript level = 1). **c** The expression of *pal1* in four parts of the *P. ostreatus* fruiting body. **d** qPCR analysis of *pal2* expression*.* The relative abundances of the transcript levels in the WT strain in the different parts of the fruiting body were normalized by comparison with that observed in the fruiting body (relative transcript level = 1). The values are the means ± SE of three independent experiments. Different letters indicate significant differences between the strains (*P* < 0.05, according to Tukey’s test)

The four stages composing the life cycle of a mushroom are the mycelium, primordia, fruiting body and spore stages (Additional file 3: Figure S3). To investigate the expression patterns of *pal1* and *pal2* during *P. ostreatus* development, the expression of these genes during different developmental stages and different parts of the fruiting bodies of the wild type (WT) strain were assessed (Fig. 3). The results showed that compared with that in mycelia, the expression of *pal1* was significantly upregulated in primordia (3.5-fold), fruiting bodies (19.3-fold) and spores (11.8-fold) (Fig. 3a). In addition, the expression of *pal2* was upregulated significantly and continuously during *P. ostreatus* development and was higher than that of mycelia in primordia (7-fold), fruiting bodies (15.2-fold), and spores (68-fold) (Fig. 3b). Figure 3c and d show *pal1* and *pal2* gene expression in different parts of the *P. ostreatus* fruiting body. The results showed that the expression of *pal1* and *pal2* in different parts of fruiting bodies had the same trend, with the highest expression observed in the gills.

### Expression of *pal1* and *pal2* and damage to mycelia under heat stress

**Fig. 4:**
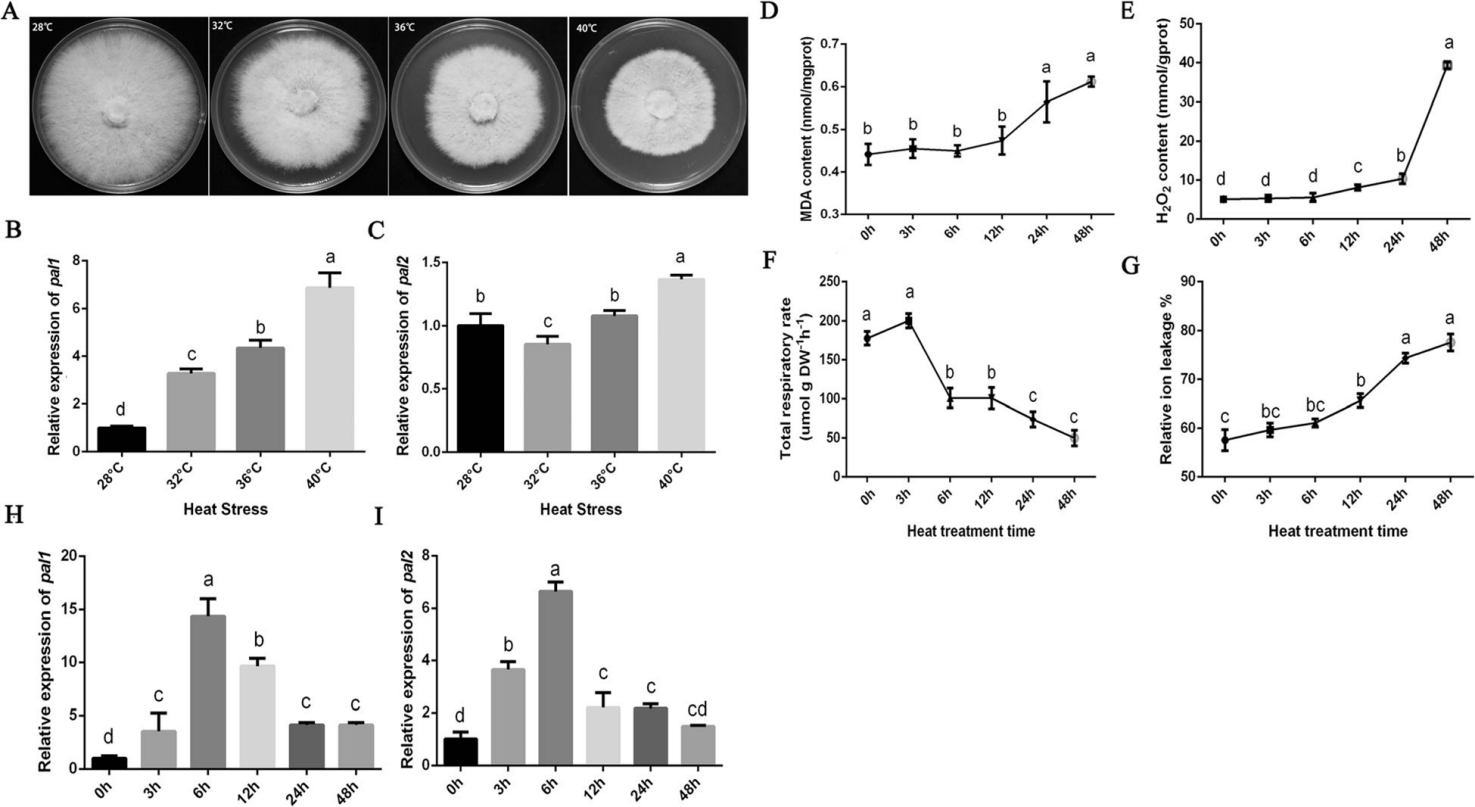
Effects of heat stress on mycelia. **a** Effects of different temperature stresses on mycelia. **b** Relative expression of *pal1* in mycelia at different temperatures. **c** Relative expression of *pal2* in mycelia at different temperatures. The relative abundances of transcripts in the mycelia at different temperatures were normalized by comparison with that observed in mycelia at 28 °C (relative transcript level = 1) (**d**) MDA content. **e** H_2_O_2_ content. **f** Total respiratory rate. **g** Relative ion leakage. **h** Relative expression of *pal1.* **i** Relative expression of *pal2.* The mean values and standard deviations of three biological replicates are shown. The error bars with different letters over the columns denote significant differences (*P* < 0.05, according to Tukey’s test)

As shown in Fig. 4a, the mycelial growth was slightly affected at 32 °C, severely inhibited at 36 °C and completely abrogated at 40 °C. The expression of *pal1* increased significantly with increasing temperature, while *pal2* expression was first downregulated and then upregulated through a small series of changes. According to the results, 40 °C was selected as the stress temperature for further study. The H_2_O_2_ and MDA content, which are two indicators of oxidative damage, increased with the duration of processing, especially when the stress time exceeded 24 h. The results showed that oxidative damage occurred in mycelia under heat stress (Fig. 4d, e). Figure 4f and g show the changes in the mycelial total respiration rate and relative ion leakage under different temperature stresses. The results showed that with increasing heat stress time, the total respiration rate of mycelia increased temporarily and then decreased rapidly. At the same time, the relative ion leakage increased significantly with increasing stress time, indicating that the degree of mycelial damage increased. Figure 4h and i show the relative expression of *pal1* and *pal2* in mycelia after different durations of heat stress. *pal1* and *pal2* expression first increased and then decreased with heat stress time. The expression of the *pal1* and *pal2* genes peaked at 6 h of heat stress.

### Generation of *pal* OE and RNAi strains

**Fig. 5:**
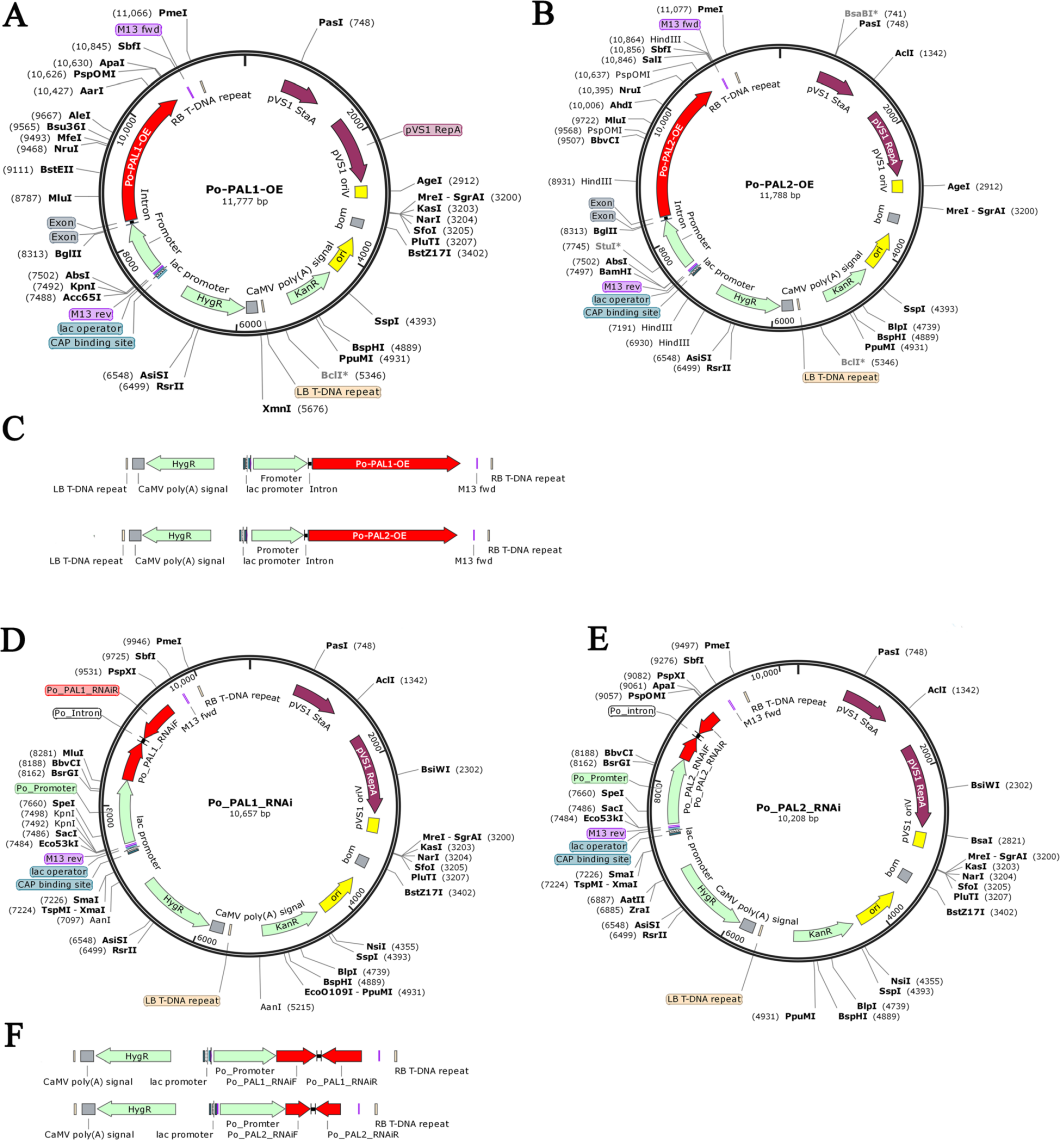
Strateg for the OE and RNAi of *pal* genes in *P. ostreatus.* **a** The structure of the *pal1* OE vector. **b** The structure of the *pal2* OE vector. **c** Schematic representation of the OE vector based on the *A. tumefaciens* right and left borders. On the left side, the *hygR* cassette is driven by the lac promoter. On the right side, the *PAL* OE cassette is driven by the *P. ostreatus gpd* promoter. **d** The structure of the *pal1* gene RNAi vector. **e** The structure of the *pal2* gene RNAi vector. **f** Schematic representation of the RNAi vector based on the *A. tumefaciens* right and left borders. On the left side, the *HygR* cassette is driven by the lac promoter. On the right side, the *pal* RNAi cassette (*pal*-RNAiF and *pal*-RNAiR) is driven by the *P. ostreatus gpd* promoter

**Fig. 6:**
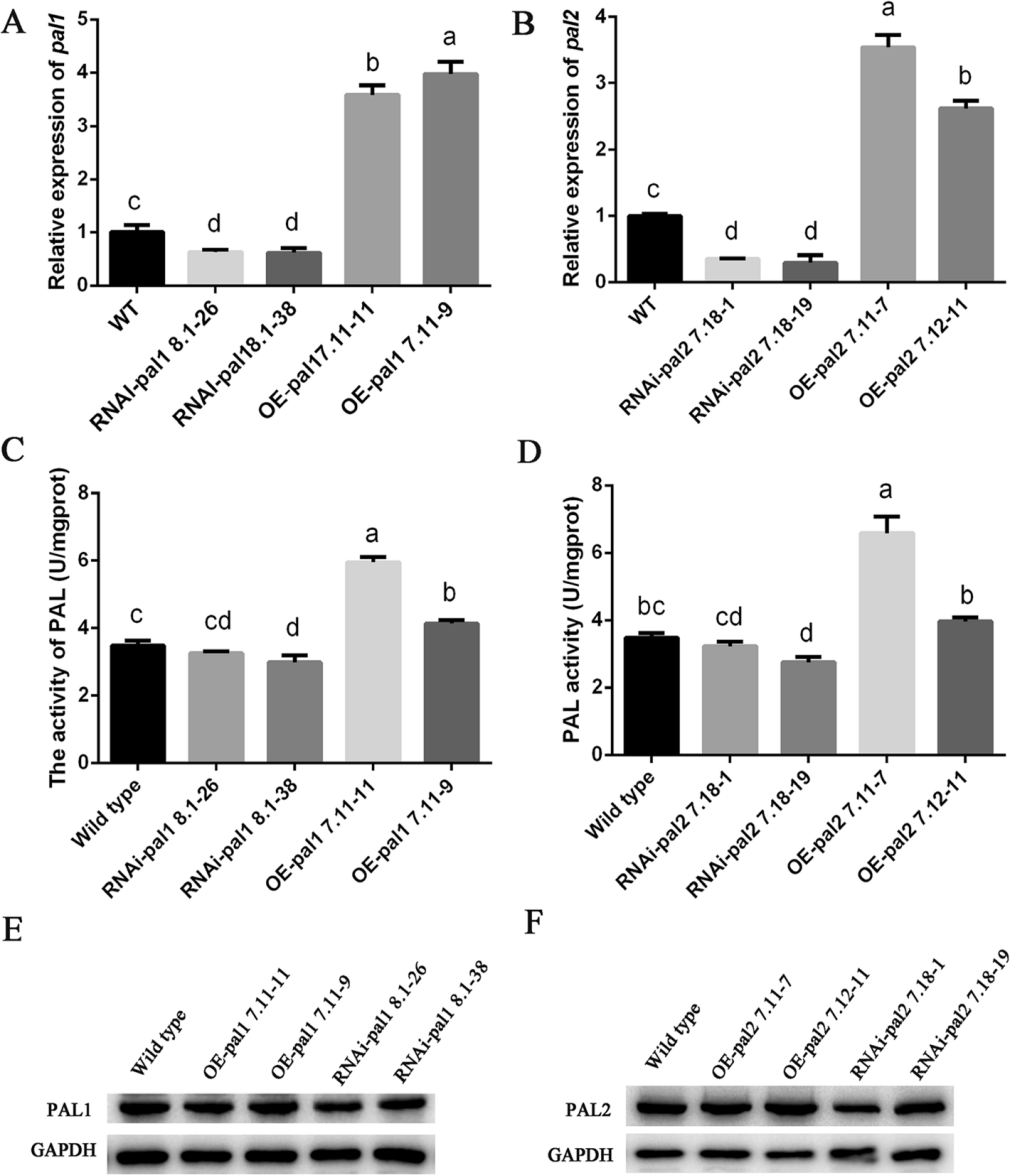
Characterization of the *pal* OE and RNAi strains. **a** qPCR analysis of the expression of *pal1* in the tested strains. **b** qPCR analysis of the expression of *pal2* in the tested strains. **c** and **d** Determination of the PAL activity in the tested strains. **e** and **f** Protein expression of *pal1* and *pal2* in the tested strains. Three independent biological replicates were performed for all experiments. The values are the means ± SE. Different letters indicate significant differences between the strains (*P* < 0.05, according to Tukey’s test)

Gene transformation with a gene knockout vector is a useful approach to explore the function of genes in fungi [31]. To study the roles of *pal1* and *pal2* in *P. ostreatus*, two RNAi-*pal* silencing vectors and two OE-*pal* OE vectors were constructed containing the *hyg* gene as a selectable marker (Fig. 5). The efficiency of RNAi and OE of the transformants was further confirmed by qPCR analysis. The transcription of *pal1* in the OE strains (OE-*pal1* 7.11–11 and OE-*pal1* 7.11–9) was approximately 4-fold higher than that of the WT strain, whereas *pal1* transcription in the RNAi strains (RNAi-*pal1* 8.1–26 and RNAi-*pal1* 8.1–38) decreased by more than 50%. Therefore, these strains were selected for further study (Fig. 6a). The transcription of *pal2* in the OE strains (OE-*pal2* 7.11–7 and OE-*pal2* 7.12–11) and RNAi strains (RNAi-*pal2* 7.18–1 and RNAi-*pal2* 7.18–19) were significantly different from that observed in the WT strain. The *pal2* gene expression of the overexpression strains was approximately 3-fold higher than that in the WT strain, while the expression in the RNAi strains decreased to 20% (Fig. 6b). The PAL enzyme activity in the tested strains was also assessed. The results showed that the PAL activity in the OE-*pal1* 7.11–11 strain was 1.7-fold greater than that in the WT strain and that the PAL activity in the OE-*pal2* 7.11–7 strain was 1.8-fold greater than that in the WT strain (Fig. 6c, d). In contrast, PAL enzyme activity was slightly decreased in the RNAi strains than that in the WT strain (Fig. 6c, d). Western blotting showed that the expression of PAL protein was slightly increased in the OE-*pal* strains, and decreased in the RNAi-*pal* strains relative to the WT strain (Fig. 6e, f).

### *pal1* and *pal2* are involved in primordium formation

**Fig. 7:**
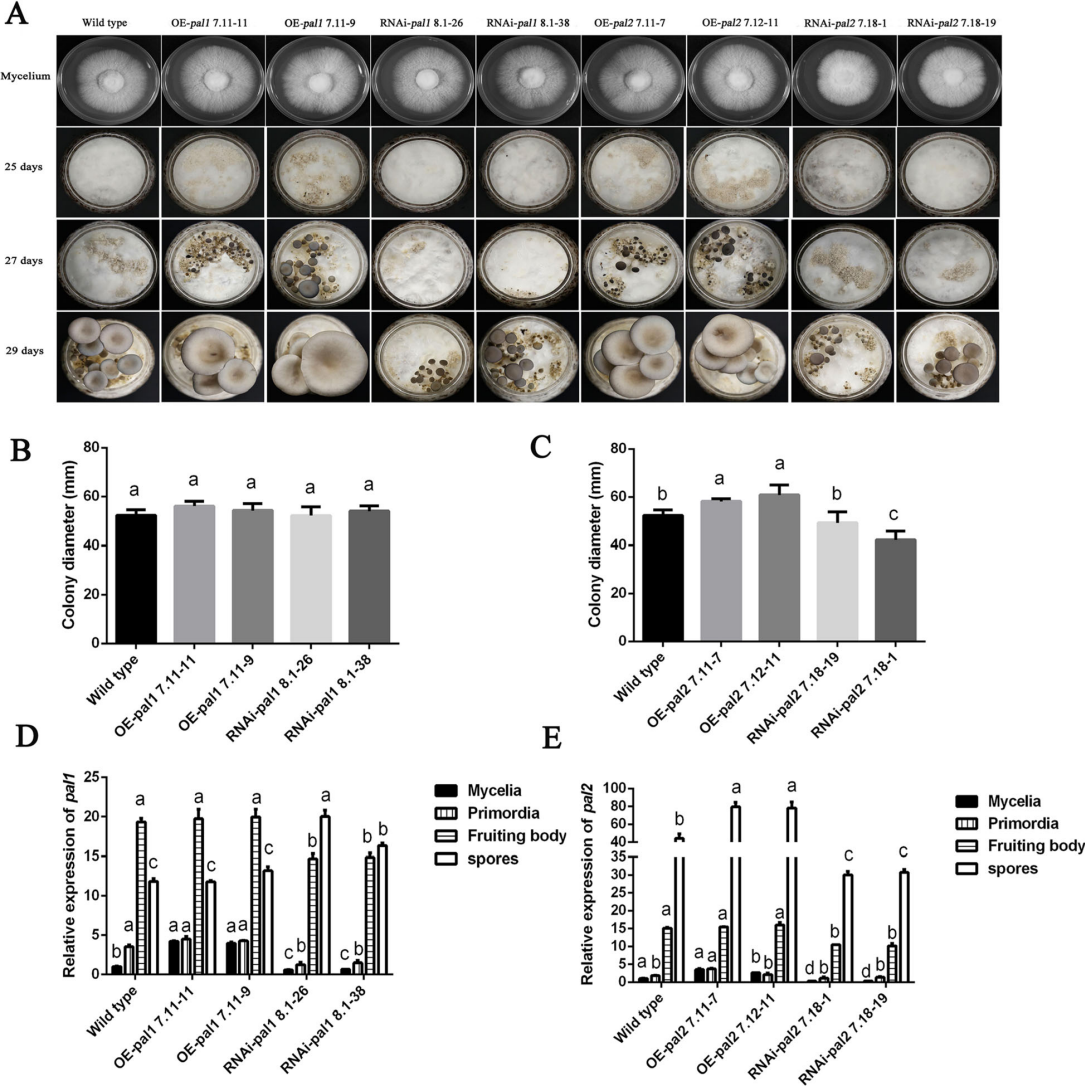
*pal1* and *pal2* are involved in the development of *P. ostreatus*. **a** Developmental stages (mycelia, primordia and fruiting body) in the life cycle of the WT, OE and RNAi strains. **b** and **c** The growth rate of the *pal* mutants. **d** and **e** qPCR analysis of *pal* expression in tested strains at different development stages (mycelia, primordia, fruiting body and spores). The relative abundances of the transcripts at different stages were normalized by comparison with that observed in mycelia in the WT strain (relative transcript level = 1). The values are the means ± SE of three independent experiments. Different letters indicate significant differences between the strains (*P* < 0.05, according to Tukey’s test)

The effects of *pal* OE and RNAi on mycelial growth are shown in Fig. 7a. The results showed that OE and RNAi of *pal1* had no visible phenotypic effects (Fig. 7a, b). However, compared with the WT strain, the OE-*pal2* strains exhibited slightly larger colony diameters (Fig. 7a), whereas the RNAi strains exhibited significantly lower growth rates (Fig. 7c). Figure 7c shows that the mycelial growth rate of the OE-*pal2* 7.11–7 and OE-*pal2* 7.12–11 strains increased by 11.21 and 16.26% respectively relative to that of the WT strain. Relative to the mycelial growth rate of the WT strain, the rates of the RNAi-*pal2* 7.18–1 and RNAi-*pal2* 7.18–19 strains were decreased by 5.82 and 19.43%, respectively. In the mushroom production experiments, we observed that OE-*pal* strains formed primordia 2 days earlier than the WT strain, whereas the RNAi strains exhibited the opposite phenotype. The results of the mushroom production experiment indicated that the time of primordium formation in the *pal* interfering strains was 2 days later than that of the WT strain (Fig. 7a). Correspondingly, the period of mushroom cultivation was shortened by *pal* overexpression and prolonged by RNAi. To further explore the biological role of *pal* in the development of fruiting bodies, the expression of *pal1* and *pal2* was assessed in the WT, OE and RNAi strains at different developmental stages by qPCR. Figure 7d and e show that the *pal* gene expression patterns in the OE-*pal* and RNAi-*pal* strains at different developmental stages were similar to those of the WT strain. *Pal* gene expression during the reproductive growth stage was higher than that during the vegetative reproductive stage except in the spores of the RNAi-*pal1* strains. In summary, *pal* overexpression can promote primordium formation, and *pal* interference can significantly prolong the time to primordium formation. OE and RNAi of *pal* have little effect on the expression patterns at different developmental stages.

### *pal1* and *pal2* participate in the regulation of the mycelial response to heat stress

**Fig. 8:**
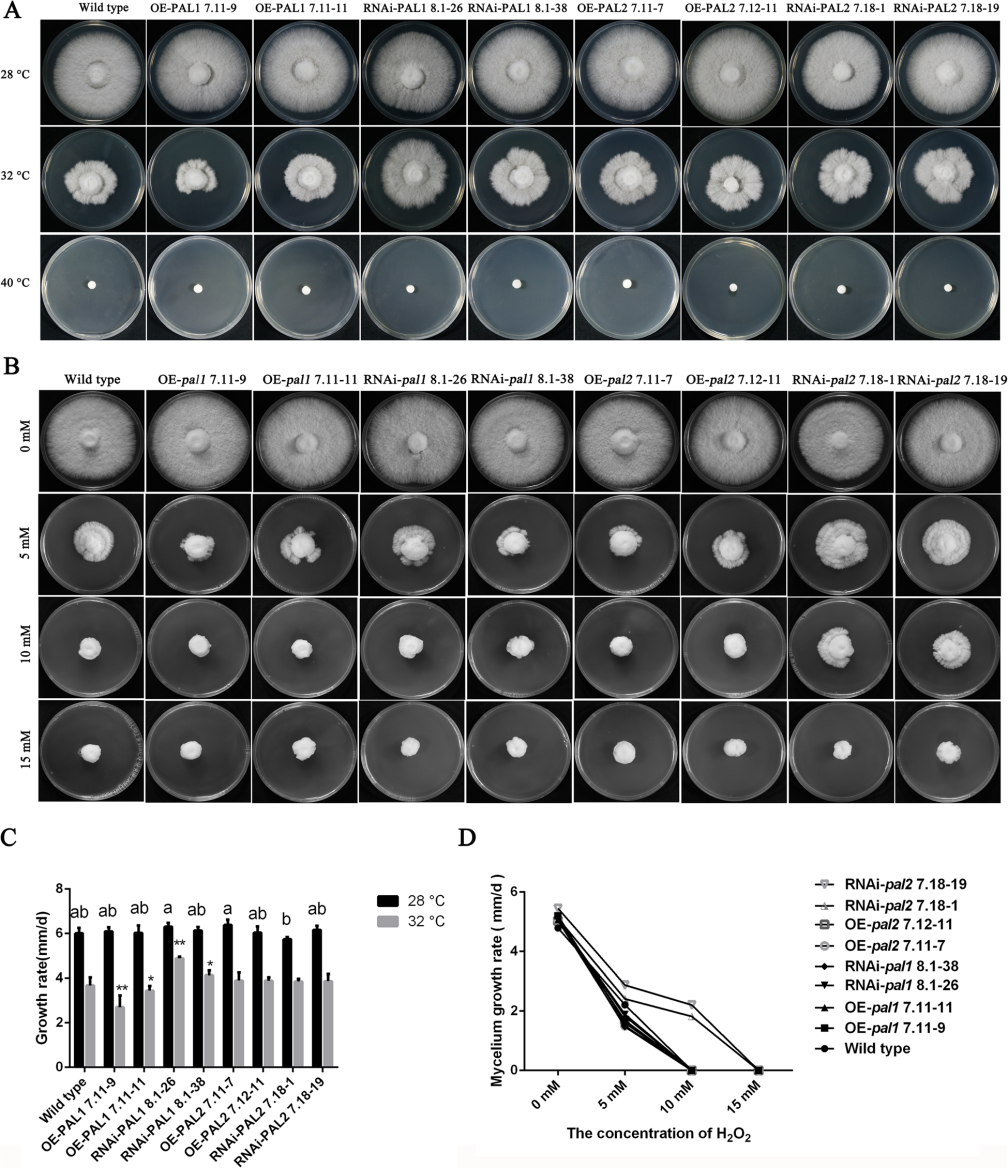
*pal1* and *pal2* participate in the resistance of mycelia to heat stress. **a** Colony morphology at different temperatures. **b** The effects of exogenous H_2_O_2_ at different concentrations on mycelial growth. **c** The growth rate of experimental strains at different temperatures. **d** Effects of different concentrations of exogenous H_2_O_2_ on the mycelial growth rate. The values are the means ± SE of three independent experiments. Different letters indicate significant differences between the transformed strains and the WT strain under 28 °C culture conditions. Different asterisks indicate significant differences between the transformed strains and the WT strain under 32 °C culture conditions. (*P* < 0.05, according to Tukey’s test)

Figure 8a and c show the growth status and rate of the tested strains. The results showed that the growth rate of the WT strain was seriously affected when the mycelial growth temperature was 32 °C, while that of the *pal1* interference strains was significantly increased compared with that of the WT strain. Similar to the WT strain, the growth rate of other tested strains was significantly inhibited, and germination actually stopped at 40 °C.

Heat stress can lead to a significant increase in H_2_O_2_ in mycelia. To investigate whether *pal* genes are involved in the reactive oxygen species (ROS) response, the growth rates of the WT, OE and RNAi strains were evaluated on PDA plates with different concentrations of exogenously added H_2_O_2_. The resistance of the RNAi-*pal2* 7.18–1 and RNAi-*pal2* 7.18–19 strains to H_2_O_2_ was higher than that of the WT strain, especially when at a H_2_O_2_ concentration of 10 mM (Fig. 8b). In addition, the resistance of other strains to H_2_O_2_ did not change significantly under the tested conditions (Fig. 8b and d). In summary, the interference of the *pal2* gene reduced the sensitivity of mycelia to H_2_O_2_.

## Discussion

PAL plays an important role in the acquisition of secondary metabolites. The role of PAL in plants has been extensively studied. However, the biological function of PAL in fungi, which has important research significance, has yet to be fully elucidated. The number and structure of *pal* genes varies greatly in different organisms, and there are several *pal* genes in fungi. In *Rhodosporidium toruloides*, *pal* is encoded by a single gene. In the genomes of *Aspergillus oryzae* RIB 40 and *Aspergillus nidulan* FGSC A4, four and two *pal* genes are encoded, respectively. In this study, two *pal* genes were identified within the *P. ostreatus* genome, which is generally consistent with that observed in other basidiomycetes. For example, two *pal* genes were identified in *Coprinopsis cinerea* and *Schizophyllum commune*. Our phylogenetic tree also supports this result. Po-PAL1 and Po-PAL2 clustered together with PALs from *Coprinopsis cinerea* and *Schizophyllum commune*, respectively. In addition, the *P. ostreatus pal1* and *pal2* genes contained 6 and 10 introns, respectively, differing greatly in genetic structure. Previous studies have shown that the number of introns in Basidiomycota *pal* genes ranges from 0 to 13 introns, and our results are consistent with these observations [32]. The nucleotide sequence consistency of *pal1* and *pal2* was 55.41%, and the amino acid consistency was 61.61% (Additional file 1 and 2: Figure S1-S2). In phylogenetic trees, the PAL1 and PAL2 sequences were not phylogenetically closely related to each other, suggesting that PAL1 and PAL2 did not undergo simple gene duplication.

PAL proteins participate in the growth and development of plants, playing different roles in different species. In *Arabidopsis*, Antje et al. reported that *pal1* and *pal2* mutants had no unique morphological phenotype but were sterile [7]. Huang et al. showed that three independent *pal1* and *pal2* double mutants generated yellow seeds due to the lack of condensed tannin pigments in the seed coat [33]. In fungi, many *pal* genes have been cloned, but little research has been performed to elucidate their biological function. In this study, the results showed that the gene expression patterns of *pal1* and *pal2* during different developmental stages were essentially the same, with *pal1* and *pal2* expression increasing during the transformation from the vegetative to the reproductive growth stages. The gene expression of each of *pal1* and *pal2* was also consistent in different parts of the fruiting body. Overexpression of *pal1* and *pal2* resulted in earlier formation of primordia, suggesting that these genes may play a similar role in the development of fruiting bodies. In *Flammulina velutipes*, the expression of *pal* in the stipe increased significantly, suggesting that *pal* may be involved in stipe elongation [31]. We observed that *pal* gene expression in the stipe was significantly lower than that in the fruiting the body, possibly because a long stipe is not a beneficial trait during *P. ostreatus* development. The transcription *pal* in the cap was significantly higher than that in the stipe. Considering that phenolic compounds in plants are synthesized through the activity of *pal* in the phenylpropanoid pathway, *pal* expression in the *P. ostreatus* cap may be involved in the production of phenolic compounds, which may improve the antioxidant capacity of mushrooms. In this study, different levels of *pal1* and *pal2* transcription were observed in spores. Previous studies have shown that among the different parts of the fruiting body of *Tricholoma matsutake*, the gill exhibits the highest transcription of *tmpal2* [34]. Our results also indicate that *pal2* transcription is significantly higher than that of *pal1* in spores, which suggests that *pal2* may play a major role in spore-related progress.

In this study, *pal1* gene expression increased significantly after heat stress, and the RNAi-*pal1* strains showed a significant growth advantage over the WT strain at 32 °C. The OE-*pal2* and RNAi-*pal2* strains showed no significant difference in growth at 32 °C but showed marked resistance to exogenous H_2_O_2_. In plants, oxidative stress is produced as a secondary stress during the heat stress response, which results in the abundant production of ROS [35]. ROS poses a serious threat to cell function by damaging lipids and proteins [36]. ROS are oxygen-containing compounds, such as superoxide anion (O_2_^−^), H_2_O_2_, and hydroxyl radicals (·OH) [37]. We speculate that *pal2* interference may reduce the sensitivity of mycelia to H_2_O_2_, whereas *pal1* interference may reduce the sensitivity of mycelia to other types of ROS. Thus, *pal1* and *pal2* may respond to stress by regulating different pathways. The results showed that *pal* may have a negative regulatory effect on the response of *P. ostreatus* to heat stress. Similar observations have been reported in plants. In *Arabidopsis thaliana*, *pal1* and *pal2* RNAi strains were more sensitive to UV-B radiation but more resistant to drought stress [33]. In *Brachypodium*, no significant difference in UV-B radiation or drought resistance was observed between RNAi*-pal* and WT plants [38].

## Conclusions

In summary, in this study, two *pal* genes were cloned, and the structural characteristics of the encoded proteins were studied. Through qPCR analysis, we observed that the gene expression patterns of *pal1* and *pal2* were essentially the same during *P. ostreatus* different developmental stages. In addition, this study confirmed that *pal* interference can delay the formation of primordia. These results indicate that *pal* genes are involved in the development of *P. ostreatus* fruiting bodies. In addition, this study assessed the role of *pal* in heat stress, providing a basis for exploring the role of the phenylpropanoid pathway in the development and stress response of *P. ostreatus.*

## Methods

### Strains, plasmids and media

The dikaryotic *P. ostreatus* strain CCMSSC00389 from the Center for Mushroom Spawn Standards and Control of China was used in this study as a parent strain for OE and RNAi experiments. The WT, OE and RNAi strains were maintained on potato dextrose agar (PDA) at 4 °C. For the selection and maintenance of transformants, complete media (CM) was supplemented with 90 μg/mL hygromycin (hyg) (Invitrogen, U.S.A.). *Agrobacterium tumefaciens* (*A. tumefaciens*) GV3101 (IMCAS, Beijing, China) was grown in Luria-Bertani (LB) medium (Oxoid, England) containing 100 μg/mL kanamycin (kan) (VWR Life Science, U.S.A.) and 50 μg/mL rifampicin (rif) (MP Biomedicals, France) and used to transform *P. ostreatus. Escherichia coli* (*E. coli*) DH5α and BL21 (DE3) (Tiangen, Beijing, China) were used for plasmid construction, and grown in LB broth containing kan (50 μg/mL). Restriction endonucleases were purchased from New England Biolabs (NEB), and DNA polymerase, a reverse-transcription kit, and a DNA Gel Extraction kit were purchased from Vazyme (Nanjing, China). Primer synthesis and DNA sequencing were performed by Sangon Biotech (Shanghai, China). The plasmid pCAMIBA 1300 was purchased from YRGen Biotech Company (Changsha, China).

### Identification, cloning and sequence analysis of *pal* genes

The two *pal* genes were identified by keyword search in the annotated genome database of *P. ostreatus* strain PC15 [39], via the Joint Genome Institute website (https://genome.jgi.doe.gov/PleosPC15_2/PleosPC15_2.home.html), and two protein sequences with the following protein IDs were obtained: 1112899 and 173,727. Subsequently, the two sequences were used to BLAST against the CCMSSC00389 genome database to identify homologs. The nucleotide sequences were used to design primers (*pal1* and *pal2*, listed in Table 1) to amplify full-length sequences from CCMSSC00389 complementary DNA (cDNA). Total RNA and DNA were extracted using TRIzol (Omega Bio-Tek, U.S.A.) and cetyltrimethylammonium bromide (CTAB), respectively. The first-strand cDNA was synthesized using a PrimeScript™ RT-PCR kit (Vazyme). The amplified products were purified and cloned into the vector pGEM-T (Promega, Madison, WI, U.S.A.) for sequencing. All primers used in the experiment are shown in Table 1.

**Table 1:** Primers used in this study

Primer	Sequence (5′ → 3′)	Note
Po-*gpd*F	GGTACCTTTATTGGCGGT	Promoter cloning
Po-*gpd*R	CCAGGTCAGTGAAATTTCC	
*pal*1_gF	ATGACAATCCTATCCGCAGAC	gDNA fragment cloning
*pal1*_gR	TTAGAATAAATCAACGATGATA	
*pal1*_cF	ATGACAATCCTATCCGCAGAC	cDNA fragment cloning
*pal1*_cR	TTAGAATAAATCAACGATGATA	
*pal2*_gF	ATGACTATTCTCTCAGGGA	gDNA fragment cloning
*pal2*_gR	CTACGCGAACATCGCTA	
*pal2*_cF	ATGACTATTCTCTCAGGGA	cDNA fragment cloning
*pal2*_cR	CTACGCGAACATCGCTA	
*pal1*-qF	CTCCTTCACAATCGCATCTA	qPCR
*pal1*-qR	CTTCAGCCGCCTATGTTG	
*pal2*-qF	CAACTGCTGCGTATGTCA	
*pal2*-qR	GATGTAGAGGTATGAGGAGATT	
*β-actin*-F	GCGATGAACAATAGCAGGG	Endogenous control
*β-actin*-R	GCTGGTATCCACGAGACAAC	
*pal1*-OE-F	ttacaggtcaaagttATGACAATCCTATCCGCAGAC	Construction of OE plasmids
*pal1*-OE-R	aattctagagggcccTTAGAATAAATCAACGATGATA	
*pal2*-OE-F	ttacaggtcaaagttATGACTATTCTCTCAGGGA	
*pal2*-OE-R	aattctagagggcccCTACGCGAACATCGCTA	
*pal1*-RNAi-F1	actgacctggGATTTGCAACCGTTGTCTTACG	Construction of RNAi plasmids
*pal1*-RNAi-R1	gttggagtgcaactccaCTAAAATCAGATGAGGTTGTAAGCG	
*pal1*-RNAi-F2	tagTGGAGTTGCACTCCAACGTGA	
*pal1*-RNAi-R2	catgccaattctagagggcccGATTTGCAACCGTTGTCTTACG	
*pal2*-RNAi-F1	aatttcactgacctggCCACCGACAATCCTCTCATCG	
*pal2*-RNAi-R1	gcacaaccaagcagtaaaCTAGAAAATGAGAATAAGACCTTGCTACC	
*pal2*-RNAi-F2	agTTTACTGCTTGGTTGTGCATTTC	
*pal2*-RNAi-R2	catgccaattctagagggcccCCACCGACAATCCTCTCATCG	
*hyg* F	CGACAGATCCGGTCGGCATCTACTCTATTTCTT	Detection of transformants
*hyg* R	TCTCGTGCTTTCAGCTTCGATGTAGGAGGG	
OE_*gpd*_*pal1*F	TGCGTGGTAGAAGAATGG	
OE_*gpd*_*pal1*R	CGATGAAGAAGGTAGAATGC	
OE_*gpd*_*pal2*F	CGTTCTCCGAGTCTGTTC	
OE_*gpd*_*pal2*R	TGATAGCGTCTTGCCATC	
*pal1*-PE-F	TCGCGGATCCGAATTCATGCAATCCTATCCGCA GAC	Construction of prokaryotic expression plasmid
*pal1*-PE-R	GAGTGCGGCCGCTTAGAATAAATCAACGATATAGGC	
*pal2*-PE-F	CGGATCCGAATTCAGACTATTCTCTAGGGACCACCG	
*pal2*-PE-R	TGCTCGAGTGCGGCCGCCTACGCAACATCGCTACCG	

### Bioinformatics analysis of the *pal* genes

DNAMAN software was used for multiple sequence alignment. The molecular weight, distribution of amino acids, isoelectric point, and signal peptide of *pal* were predicted using the online ProtParam (http://web.expasy.org/protparam/). The structural domains of the PAL proteins were analyzed online (http://smart.embl-heidelberg.de/). A phylogenetic tree was constructed using the neighbor joining method in MEGA 5.0 based on the PAL nucleotide sequences obtained from GenBank and the maximum composite likelihood model. The three-dimensional (3D) structure of the PAL proteins were predicted using Modeller.

### Expression and purification of *pal1* and *pal2* in *E. coli*

Expression and purification of *pal1* and *pal2* in *E. coli* were performed as previously described with slight modifications [40]. The PCR products were digested with two restriction enzymes (*Eco*RI-HF and *Not*I-HF) and ligated into the vector pET28a (Novagen, Inc., Madison, WI, U.S.A.) that had been digested with the same enzymes. The recombinant plasmids that were confirmed by DNA sequencing were named pET28a-*pal1* and pET28a-*pal2* and were subsequently transformed into *E. coli* BL21 (DE3) cells for protein expression. The transformed strains were inoculated into LB medium containing 50 μg/mL kan and incubated at 37 °C with shaking at 180 rpm until reaching an OD600 nm of 0.6–0.8. Isopropyl-β-D-thiogalactopyranoside (IPTG) was added to reach a final concentration of 1 mM to induce protein expression, and the culture was incubated overnight at 16 °C with shaking at 180 rpm.

The cultured cells were centrifuged at 4 °C and 5000 rpm for 5 min, washed with PBS buffer, and then suspended in lysis buffer. After the cells were lysed by ultrasonication, the enzymes were retained in the supernatant after centrifugation. The supernatant was loaded onto an Ni-NTA column (Qiagen, Duesseldorf, Germany) that had been preequilibrated with binding buffer. Subsequently, the column was eluted with binding buffer, washing I buffer, washing II buffer, elution I buffer and elution II buffer. Finally, the fractions were analyzed by sodium dodecyl sulfate-polyacrylamide gel electrophoresis (SDS-PAGE) [41]. The PAL activities of the purified protein were determined using a Phenylalanine Ammonia-Lyase Assay kit (Nanjing Jiancheng Bioengineering Institute, Nanjing, China) according to the manufacturer’s instructions.

### OE and RNAi vector construction

The original pCAMBIA1300 vector was modified to harbor the hyg phosphotransferase gene (*hyp*), which was expressed under the control of the upstream lac promoter [28, 42]. The *pal* gene OE cassettes were constructed as follows. The *P. ostreatus gpd* promoter was PCR amplified, after which the *pal1* and *pal2* cDNA was obtained. The two cassettes were individually cloned into vector to generate the *pal* gene expression cassette driven by the *P. ostreatus gpd* promoter (Fig. 5a, b, c). Finally, the vector was introduced into *A. tumefaciens* GV3101. RNAi-F and RNAi-R fragments were obtained by PCR, after which the two amplicons were individually inserted into the vector to construct the interference vectors (Fig. 5d, e, f). Finally, the interference vectors were transferred into *P. ostreatus* by *A. tumefaciens* GV3101. The primers used to construct the vectors are shown in Table 1.

### Agrobacterium-mediated transformation

*P. ostreatus* mycelia were inoculated onto PDA plates and cultured at 28 °C until the colony diameter was 5.5–6 cm. Mycelial pellets were cut from the edge of the colony using a cork borer with a 5-mm diameter. Subsequently, 200 pellets were placed into 100 mL of CM liquid medium at 28 °C for 2 days without shaking. *A. tumefaciens* GV3101 containing the OE-*pal* or RNAi-*pal* plasmid was cultivated at 28 °C with shaking at 180 rpm in LB medium with the selective antibiotics (100 μg/mL kan and 50 μg/mL rif) for at least 16 h. *A. tumefaciens* cells were collected in sterile tubes (50 mL capacity) by centrifugation at 4500 rpm and 4 °C for 10 min. The bacterial cells were suspended in induction medium (IM,; supplemented with 200 μM acetosyringone) and incubated for 5 h (90 rpm, 28 °C) to preinduce *A. tumefaciens* GV3101 virulence. Then, the *A. tumefaciens* GV3101 and the mycelia pellets were cocultured at 28 °C for 5 h without shaking. After incubation, the mycelial pellets were dried with filter paper and placed onto IM solid medium at 28 °C for 3 days and then transplanted onto CM medium with selective antibiotics (90 μg/mL hyg and 50 μg/mL cef). Transformants were obtained after 25 days of culturing and were subsequently selected twice for hyg resistance. PCR analysis of the *hyg* and *pal* genes was performed using the primers listed in Table 1 [42].

### Mushroom production experiment and sample collection

The strains were cultured on PDA plate for 7 days. Samples mycelia were collected, frozen with liquid nitrogen and stored at − 80 °C. The remaining mycelia were inoculated on cotton seed hull culture medium and cultured at 25 °C in dark. When the mycelia were full, they were transferred to a mushroom room for mushroom production. The primordia, fruiting bodies and spores were collected and stored at − 80 °C after quick freezing with liquid nitrogen.

### Western blot analysis

Western blot analysis was performed according to a previous study. Briefly, equal amounts of total protein (20 μg) were loaded into the protein lane and separated in a 12% (w/v) SDS-PAGE. After electrophoresis, proteins were transferred onto a polyvinylidene fluoride membrane. Western blot analysis was performed using antibodies against PAL1 and PAL2, which were synthesized by Shanghai GenScript Company. Glyceraldehyde 3-phosphate dehydrogenase (GAPDH, PC15_1090663 (jgi)) was used as control [19].

### Heat stress treatment

WT, *pal*-overexpressing (OE-*pal*1 7.11–9, OE-*pal*1 7.11–11, OE-*pal*2 7.11–7 and OE-*pal*2 7.12–11) and RNAi transformant strains (RNAi-*pal*1 8.1–26, RNAi-*pal*1 8.1–38, RNAi-*pal*2 7.18–1 and RNAi-*pal*2 7.18–19) were used in this study. The WT strain was cultured on PDA plates incubated at 28 °C for 5 days and then subjected to different temperatures to induce heat stress for 2 days. To assess the function of *pal* in the mycelial response to heat stress, the WT, OE-*pal* and RNAi-*pal* strains were cultured on PDA plates at different temperatures (28, 32, and 40 °C) for 6 days [19, 43].

### Growth susceptibility assay

To assess the susceptibility of the WT, OE-*pal* and RNAi-*pal* strains to oxidative stress, mycelial tip pellets with 5-mm diameter were inoculated onto PDA plates supplemented with 5, 10 or 15 mM H_2_O_2_. The control groups were subjected to the nonexogenous addition of H_2_O_2_. The diameters of the strains were measured after incubation at 28 °C for 7 days [44].

### Quantitative real-time PCR (qPCR)

To analyze the expression of *pal* at different developmental stages, samples were collected at the mycelia, primordia, fruiting body and spore stages. The levels of gene-specific mRNA expressed by the WT, OE-*pal* and RNAi-*pal* strains were analyzed using qPCR according to our previous study [19], with the *β-actin* gene used as a reference. The qPCR amplification procedure was as follows: 95 °C for 3 min, 40 cycles of 95 °C for 3 s and 60 °C for 32 s, and a final extension at 72 °C for 30 s. The relative gene expression was analyzed according to the 2^−△△CT^ method.

### Enzymatic activity assay

The WT, OE-*pal* and RNAi-*pal* strains were cultured on PDA medium incubated at 28 °C for 5 days. Subsequently, the mycelia were quickly scraped, mixed, and frozen in liquid nitrogen for further use. The activity of PAL was determined using a Phenylalanine Ammonia-Lyase Assay kit according to the manufacturer’s instructions.

### Determination of malondialdehyde (MDA) and H_2_O_2_ contents

Intracellular MDA and H_2_O_2_ contents were determined using a Malondialdehyde and Hydrogen Peroxide Assay kit (Nanjing Jiancheng Bioengineering Institute, Nanjing, China) according to the manufacturer’s instructions.

### Determination of relative ion leakage and total respiratory rate

Ten pellet pieces (5 mm) were inoculated into 100 mL of potato dextrose broth medium for 5 days at 28 °C with shaking at 180 rpm. Heat stress was then applied for different durations at 40 °C (0, 3, 6, 12, 24, and 48 h). The conductivity of mycelial pellets (C1) was measured by washing off the electrolytes attached to the surface with deionized water and then placing the pellets them into 20 mL of deionized water at 28 °C for 2 h. Then, the sample was autoclaved for 30 min to determine the total conductivity (C2). The relative ion leakage rate (%) = C1/C2*100 [45]. The respiration rate was determined by measuring the production of carbon dioxide with a carbon dioxide meter (MultiRAE IR PGM-54) in sealed containers. The total respiratory rate was measured according to previous studies [22].

### Data analysis

GraphPad Prism 6 (GraphPad Software, Inc., San Diego, CA, U.S.A.) was used for statistical analysis. The values are reported as the means ± SE and were analyzed by one-way ANOVA, with a *P* value of < 0.05 considered significant.

## Supplementary information

**Additional file 1: Figure S1.** Amino acid sequence alignment. (A) PAL1 of CCMSSC00389 and PC15. (B) PAL2 of CCMSSC00389 and PC15. (C) PAL1 and PAL2 of CCMSSC00389.

**Additional file 2: Figure S2.** Nucleotide sequences alignment. (A) *pal1* of CCMSSC00389 and PC15. (B) *pal2* of CCMSSC00389 and PC15. (C) *pal1* and *pal2* of CCMSSC00389.

**Additional file 3: Figure S3.** Different developmental stages of *P. ostreatus* CCMSSC00389. (A) Mycelia. (B) Primordia. (C) Fruiting body. (D) Spores (The red arrow points to spores).

## Data Availability

The datasets used and analysed during the current study available from the corresponding author on reasonable request.

## Abbreviations

cDNA: Complementary DNA
CM: Complete media
CTAB: Cetyltrimethylammonium bromide
hyg: Hygromycin
*hyp*: Hygromycin phosphotransferase gene
IM: Induction medium
IPTG: Isopropyl-β-D-thiogalactopyranoside
kan: Kanamycin
LB: Luria-Bertani
MDA: Malondialdehyde
MIO: 4-methylideneimidazole-5-one
OE: Overexpression
PAL: Phenylalanine ammonia-lyase
*pal*: Phenylalanine ammonia-lyase gene
PDA: Potato dextrose agar
pI: Isolectric point
qPCR: Quantitative real-time PCR
rif: Rifampicin
RNAi: RNA interference
ROS: Reactive oxygen species
SDS-PAGE: Sodium dodecyl sulfatepolyacrylamide gel electrophoresis
WT: Wild type
