# Supplementary figures and images for: Expression patterns of two pal genes of Pleurotus ostreatus across developmental stages and under heat stress
Source: BMC Microbiol. 2019 Oct 26;19:231. doi: 10.1186/s12866-019-1594-4 (PMC6815457; doi:10.1186/s12866-019-1594-4)

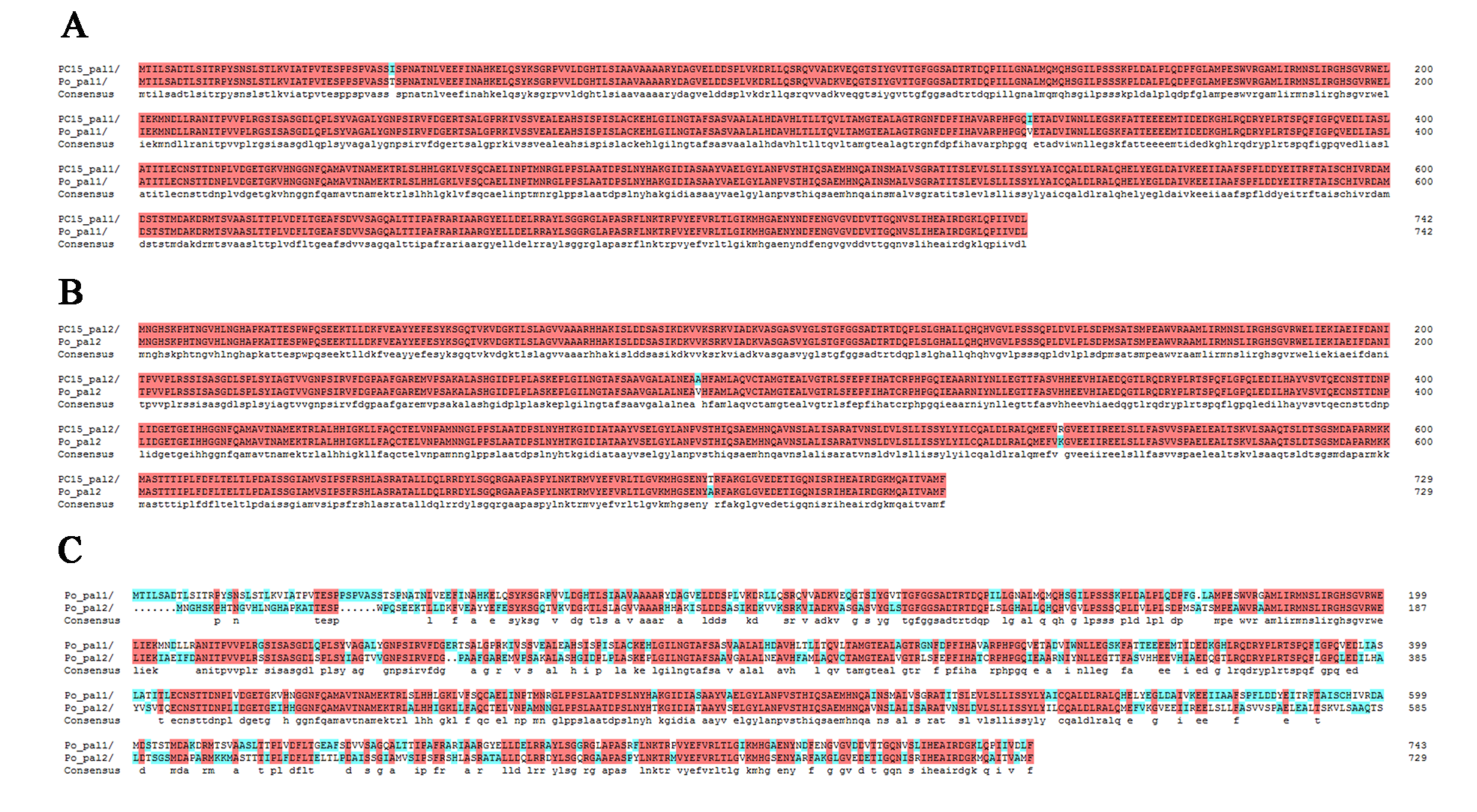

Supplement: Supplementary file 1 — Additional file 1: Figure S1. Amino acid sequence alignment. (A) PAL1 of CCMSSC00389 and PC15. (B) PAL2 of CCMSSC00389 and PC15. (C) PAL1 and PAL2 of CCMSSC00389. [file 12866_2019_1594_MOESM1_ESM.tif]

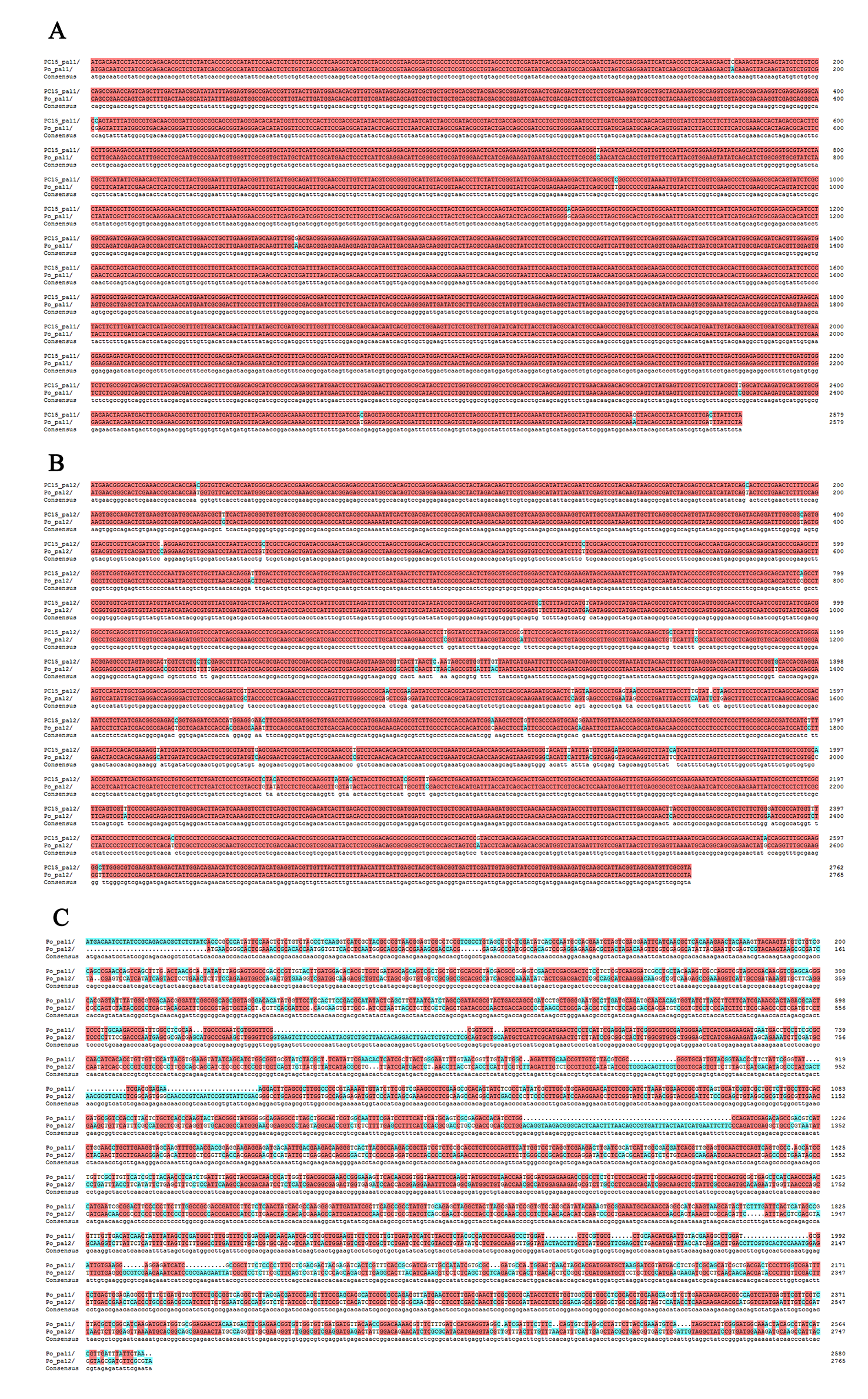

Supplement: Supplementary file 2 — Additional file 2: Figure S2. Nucleotide sequences alignment. (A) pal1 of CCMSSC00389 and PC15. (B) pal2 of CCMSSC00389 and PC15. (C) pal1 and pal2 of CCMSSC00389. [file 12866_2019_1594_MOESM2_ESM.tif]

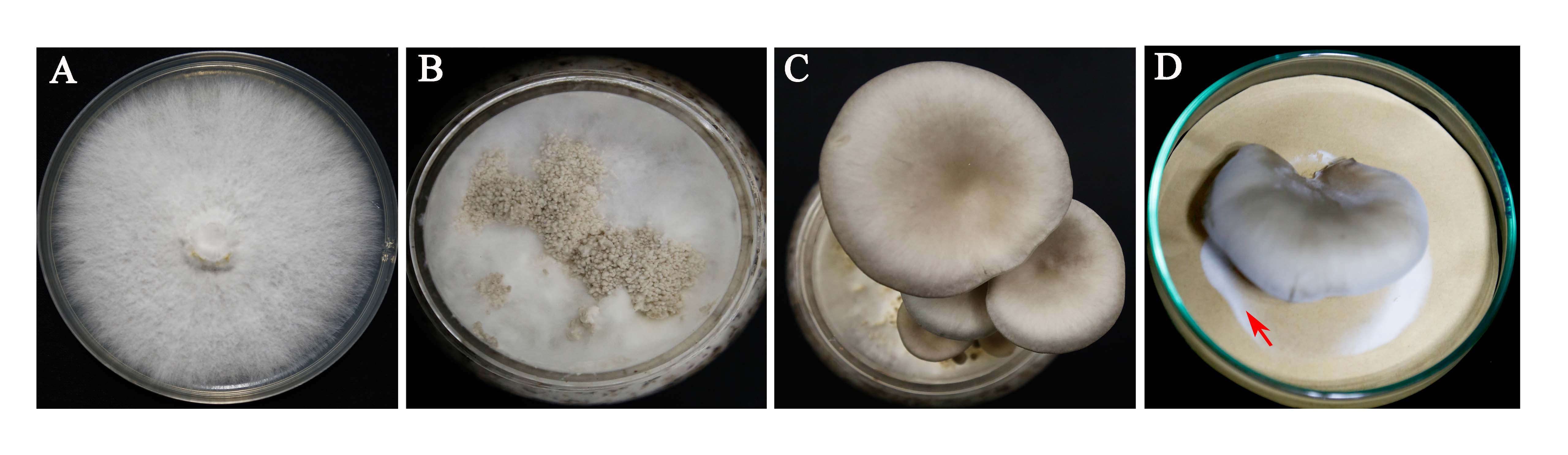

Supplement: Supplementary file 3 — Additional file 3: Figure S3. Different developmental stages of P. ostreatus CCMSSC00389. (A) Mycelia. (B) Primordia. (C) Fruiting body. (D) Spores (The red arrow points to spores). [file 12866_2019_1594_MOESM3_ESM.tif]
